# The complete mitochondrial genome of the estuarine amphipod *Grandidierella osakaensis* (Crustacea: Amphipoda)

**DOI:** 10.1080/23802359.2020.1778559

**Published:** 2020-09-08

**Authors:** Kyoshiro Hiki, Hiroyuki Ariyama, Nobuyoshi Nakajima

**Affiliations:** a Center for Environmental Risk Research, National Institute for Environmental Studies, Tsukuba, Japan; b Osaka Museum of Natural History, Osaka, Japan; c Center for Environmental Biology and Ecosystem Studies, National Institute for Environmental Studies, Tsukuba, Japan

**Keywords:** Amphipoda, Aoridae, *Grandidierella*, mitochondria

## Abstract

The complete mitochondrial genome sequence of an estuarine amphipod *Grandidierella osakaensis* was determined. The mitochondrial genome was 14,658 bp in length with 37 mitochondrial genes (13 protein-coding genes [PCGs], 2 ribosomal RNAs [rRNAs], and 22 transfer RNAs [tRNAs]). The order of PCGs of *G. osakaensis* was identical to those of other two *Grandidierella* species. A maximum likelihood-based phylogenetic analysis showed that *G. osakaensis* formed a monophyletic clade with the other two *Grandidierella* species within the infraorder Corophiida. The mitochondrial genome sequence obtained in this study provides useful information for further phylogenetic and ecological studies.

The genus *Grandidierella* Coutière, 1904 is a group of amphipods including more than 40 described species (Horton et al. 2020). The species of this genus inhabit mainly brackish benthic environments and are distributed worldwide. Several species of this genus resemble well to each other (Ariyama and Taru 2017; Myers et al. 2019), making morphological species identification challenging. In addition, the presence of cryptic species is suspected for a species of this genus (Pilgrim et al. 2013). To address these problems, molecular characterization would be useful. However, only two mitogenomes of the genus *Grandidierella* have been published (Hiki et al. 2020). In this study, we sequenced and analyzed the complete mitochondrial sequence of *G. osakaensis* (Ariyama 1996). The obtained complete mitogenome will be useful for future phylogenetic and ecological studies on the genus *Grandidierella*.

The specimens of *Grandidierella osakaensis* were collected from the intertidal zone at Ebisu-zaki in Wakayama, Japan (34°18′26ʺN, 135°04′41ʺE) on 20 April 2019. DNA was extracted using DNeasy Blood and Tissue Kit (Qiagen, Hilden, Germany) and sequenced on an Illumina MiSeq with a paired-end library. The extracted DNA was deposited in the Radioisotope & Biotechnology Laboratory at the National Institute for Environmental Studies, Japan (NIES-202002-AMP3). The raw reads were filtered using Trimmomatic version 0.36 with the following parameters: LEADING: 20, TRAILING: 20, SLIDINGWINDOW:4:20, and MINLEN:50. The processed reads were *de novo* assembled into the mitochondrial genome sequence using GetOrganelle version 1.6.4 (Jin et al. 2019) with ‘animal_mt’ in the default database as seed reads. The obtained assembly graph was visually checked by Badage version 0.8.1 (Wick et al. 2015). The mitogenome was annotated by MITOS 2 webserver (Bernt et al. 2013) and by manual comparison with orthologous genes of other amphipod species. Phylogenetic analyses were performed based on 13 protein-coding genes (PCGs) sequences in the mitogenome of *G. osakaensis* and those of other amphipods belonging to the infraorder Corophiida. Each amino acid sequence was aligned separately using MAFFT version 7.453 with the L-INS-i option (Katoh and Standley 2013) and then the alignments were filtered using trimAl version 1.4.1 (Capella-Gutiérrez et al. 2009) with the heuristic method. Maximum likelihood-based phylogenetic trees were inferred using IQ-TREE version 1.6.12 (Nguyen et al. 2015).

The complete circular mitogenome of 14,658 bp was obtained for *G. osakaensis* (INSDC accession number: LC546828) with 123× mean coverage (SD: 37, minimum: 20, maximum: 242), indicating highly confident assemblies. The mitogenome was enriched in A and T nucleotides (70.9%) and contained typical gene components, including 13 PCGs, 2 ribosomal RNAs (rRNAs), and 22 transfer RNAs (tRNAs). Of these 37 genes, 24 genes located on the light (forward) strand, while 13 located on the heavy strand. The PCG order (*cox1*, *cox2*, *atp8*, *atp6*, *cox3*, *nad3*, *nad6*, *nad5*, *nad4*, *nad4l*, *cytb*, *nad1*, and *nad2*) was the same as those in other two *Grandidierella* species, *G. rubroantennata* and *G. fasciata* (Hiki et al. 2020), but had translocations of nad6 from pancrustacean ground pattern (Boore 1999). There were three types of start codons for PCGs: ATT (*cox1*, *atp8*, *nad6*, *nad4l*, *nad1*, and *nad2*), ATA (*cox2* and *atp6*), and ATG (*cox3*, *nad3*, *nad5*, *nad4*, and *cytb*). Twelve PCGs ended with TAA as stop codon and one (*nad4l*) ended with TAG. Phylogenetic analyses showed that *G. osakaensis* was grouped with the other two *Grandidierella* species with a high bootstrap value (Figure 1) and these three species formed a monophyletic clade within the infraorder Corophiida.

**Figure 1.**
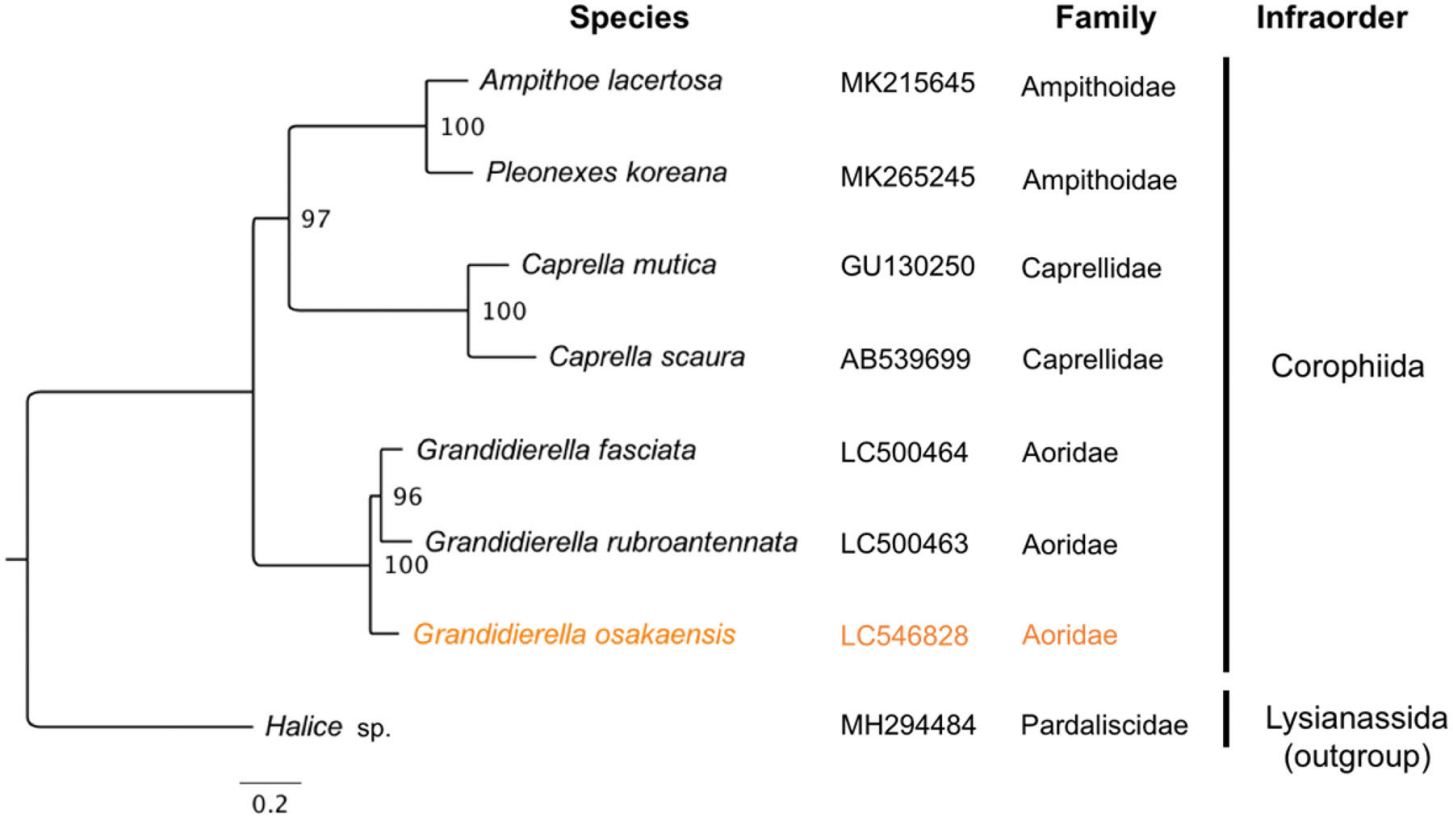
Maximum likelihood tree based on 13 PCGs in mitochondrial genomes of amphipod species belonging to the infraorder Corophiida. Orange represents the genome obtained in this study. Non-parametric bootstrap values (based on 2000 times resampling) are shown at nodes. The phylogenetic tree was inferred using IQ-TREE version 1.6.12 with the “–spp” option to allow partition-specific evolution rates. The best-fit model for each PCG was determined by ModelFinder (Kalyaanamoorthy et al. 2017) implemented in IQ-TREE, based on Akaike information criteria. The phylogenetic tree was visualized by FigTree version 1.4.4.

## Data Availability

Data that support the findings of this study are openly available in Genbank (https://www.ncbi.nlm.nih.gov/) with the accession number LC546828.
